# Physical Fitness and Cognitive Performance in Preschool Children: Cross‐Sectional and Longitudinal Associations From the ELFIT Trial

**DOI:** 10.1111/sms.70325

**Published:** 2026-06-22

**Authors:** Carlos Martin‐Martinez, Pedro L. Valenzuela, Asier Mañas, Oscar Martinez‐de‐Quel

**Affiliations:** ^1^ Department of Physiotherapy, Occupational Therapy, Rehabilitation and Physical Medicine Universidad Rey Juan Carlos Madrid Spain; ^2^ Actividad Física y Salud (AFYS) Research Group Universidad Rey Juan Carlos Madrid Spain; ^3^ Move4Life Research Group Public University of Navarre (UPNA) Tudela Spain; ^4^ Department of Health Sciences Public University of Navarre (UPNA) Tudela Spain; ^5^ Department of Systems Biology University of Alcalá Madrid Spain; ^6^ CIBER de Fragilidad y Envejecimiento Saludable, CIBERFES Instituto de Salud Carlos III Madrid Spain; ^7^ Faculty of Education, Psychology and Sport Sciences University of Huelva Huelva Spain

**Keywords:** early childhood education, executive function, muscular strength, neurodevelopment, physical activity

## Abstract

Growing evidence shows that physical fitness and cognitive function might be interrelated, but evidence is mostly cross‐sectional and research in preschool children remains scarce. We aimed to analyze both cross‐sectional and longitudinal associations between fitness indicators and cognitive outcomes in preschoolers. In this secondary analysis of the ELFIT trial, 99 children (58 girls; mean age 4.8 ± 0.7 years) were assessed at baseline and after a 10‐week intervention. Physical fitness (cardiorespiratory fitness, speed‐agility, handgrip strength, and standing long jump) was measured with the PREFIT battery, whereas cognitive outcomes were assessed using selected computerized BENCI subtests. Cross‐sectional analyses revealed significant associations of cardiorespiratory fitness with verbal memory, delayed verbal memory, cued recall, and verbal fluency, whereas handgrip strength was associated with free recall, and speed‐agility and lower‐limb strength with verbal comprehension (figures) (all pFDR < 0.05). In longitudinal analyses, improvements in cardiorespiratory fitness were significantly associated with improvements in free recall (pFDR = 0.009). Improvements in handgrip strength were associated with free recall (pFDR = 0.009), cued recall (pFDR = 0.010), verbal memory (pFDR = 0.009), delayed verbal memory (pFDR = 0.042), and delayed verbal memory recognition (pFDR = 0.011). Speed‐agility was longitudinally associated with improvements in free recall (pFDR = 0.010), verbal memory (pFDR = 0.010), and verbal fluency (pFDR = 0.049). Lower‐limb strength was associated with free recall (pFDR = 0.026), verbal memory (pFDR = 0.009), and verbal fluency (pFDR = 0.049). No significant associations were observed for reaction time or visuomotor outcomes after FDR correction. Overall, these findings suggest that fitness–cognition relationships in preschool children may be selective rather than generalized across cognitive domains, with the most consistent associations involving memory‐ and language‐related outcomes.

## 1 Introduction

The preschool years represent a critical period for brain development, characterized by rapid gains in cognitive, motor, and socio‐emotional domains [1]. The foundations of executive function, memory, and attention established during this stage are pivotal, acting as foundational predictors of future academic achievement and lifelong well‐being [2, 3]. Concurrently, the development of physical fitness in early childhood is not only crucial for physical health but also lays the groundwork for a physically active lifestyle [4].

A growing body of evidence suggests that physical fitness may be associated with cognitive performance from an early age [5, 6, 7]. Physical activity may contribute to these associations, at least in part, through its positive effects on physical fitness, which has been proposed as one of the mechanisms linking movement behaviors with cognitive development. Recent studies have reported cross‐sectional associations between cardiorespiratory fitness and executive function outcomes in preschool children (aged 3–6 years) [8, 9]. Several plausible mechanisms have been proposed, including activity‐dependent neuroplasticity, increased cerebral blood flow, and the expression of neurotrophic factors such as brain‐derived neurotrophic factor (BDNF), which may contribute to neuronal growth and synaptic connectivity [10]. Indeed, previous preschool studies and movement‐based intervention programs have reported associations between physical activity, motor skills, and selected cognitive outcomes during early childhood [8, 11].

Despite this biological plausibility and the promising results observed in intervention studies, most evidence derives from school‐aged populations and cross‐sectional studies. While valuable, these designs offer a static “snapshot” that limits our understanding of how this relationship evolves over time. For instance, a recent cross‐sectional study by Zhou et al. [9] reported significant associations between various physical fitness components (such as cardiorespiratory fitness and speed‐agility) and executive functions in preschool children. Similarly, Li et al. [12] observed that higher agility and coordination were cross‐sectionally associated with better inhibitory control in preschool children, while Niederer et al. [1] showed both cross‐sectional and longitudinal associations between aerobic fitness, motor skills, and memory/attention in children aged 5–6 years. More recently, García‐Alonso et al. [13] reported that physical fitness was positively related to executive function in preschoolers. Together, these studies indicate that associations between physical fitness and cognitive function may already emerge during the preschool years. Nevertheless, the extent to which these associations differ across developmental stages remains unclear, and evidence is particularly limited in early childhood, with most available studies being cross‐sectional; this underscores the need for further longitudinal research during the preschool years.

Consequently, there is scarce longitudinal research examining whether changes in physical fitness are associated with concurrent changes in cognitive performance. Such an approach is critical as it moves beyond static associations to provide more robust evidence for the dynamic and potentially directional nature of this relationship [14]. To address this gap, the present study examined both cross‐sectional and longitudinal associations between distinct components of physical fitness—cardiorespiratory capacity, muscular strength, and speed‐agility—and cognitive performance in preschool children. By focusing on this underexplored age group and incorporating dynamic measures over time, our study provides novel evidence on the potential role of fitness in early cognitive development.

## 2 Material and Methods

### 2.1 Study Design

The present study is a secondary analysis of the English Learners Fit (ELFIT) trial (more details in Martin‐Martinez et al. [15]). The ELFIT trial was a single‐center, cluster‐randomized controlled study carried out in Madrid, Spain, between January and March 2023. The primary study aimed to assess the effects of physically active foreign language lessons on cognitive and fitness outcomes in preschoolers. The intervention was delivered for 10 weeks during English‐as‐a‐foreign‐language sessions (2 × 45 min/week), with four pre‐established classrooms randomly allocated to either a control group (standard sedentary instruction) or one of two intervention groups incorporating light (LPA) or moderate‐to‐vigorous physical activity (MVPA). However, in the present secondary analysis, we examined the entire sample of participants—regardless of group assignment—to explore both cross‐sectional and longitudinal associations between cognitive outcomes and physical fitness measures. The study was approved by the Research Ethics Committee of Universidad Complutense de Madrid (Ref: CE_20211118‐16_SAL).

### 2.2 Participants

Children aged 3–6 years were recruited from four classrooms in a single school using a convenience sample. Parents or legal guardians of all potential participants provided written informed consent prior to the study. Eligibility criteria included the ability to engage in physical activity and complete cognitive assessments. Exclusion criteria comprised medical or neurodevelopmental conditions contraindicating physical activity or cognitive testing, as well as prior exposure to English beyond the school setting. To ensure inclusiveness, all students in participating classrooms took part in the activities, whereas research assessments and analyses were conducted only among children with parental or legal guardian consent.

### 2.3 Outcomes

Assessments were conducted at baseline and after the 10‐week intervention. A blinded external examiner, assisted by classroom teachers, administered all assessments both at baseline and post‐intervention. Cognitive assessments were administered individually using the BENCI battery [16] on a tablet (iPad 6 9.7″ screen, Apple, CA). Although the complete BENCI battery has not been formally validated for Spanish‐speaking preschool children aged 3–6 years, the selected subtests and administration parameters used in the present study were applied under standardized and developmentally adapted conditions for exploratory use in preschool populations. The selected subtests included verbal memory, delayed verbal memory, delayed verbal memory recognition, verbal comprehension (images and figures), verbal fluency, reaction time, visuomotor skills, and alternate visuomotor skills. Primary analyses focused on visuomotor time‐based outcomes, whereas exploratory error‐based visuomotor outcomes were additionally examined in supplementary sensitivity analyses. Assessments were individually administered with visual support and examiner guidance adapted to preschool developmental level. English vocabulary retention was measured through free‐ and cued‐recall tests [11, 17, 18], involving the English vocabulary target words.

Physical fitness was comprehensively assessed using the PREFIT battery, which has established validity and reliability for this age group [19], including anthropometry, handgrip and standing long jump (muscular strength), 4 × 10‐m shuttle run (speed‐agility), and the 20‐m shuttle run (cardiorespiratory fitness). All tests were administered by trained researchers following standardized protocols.

For some outcomes, lower values indicated better performance (i.e., completion time in the 4 × 10‐m shuttle run [speed‐agility], reaction time, visuomotor and alternate visuomotor time and errors), whereas for the remaining tests, higher values indicated better performance. Therefore, regression coefficients were interpreted according to the directionality of each specific outcome.

Potential covariates included age, sex, baseline height and baseline weight, socioeconomic status assessed on a scale from 1 to 5 (i.e., low, medium‐low, medium, medium‐high, and high) with the BENCI questionnaire, and out‐of‐school physical activity as reported by the parents (assessed through the parent questionnaire by Bacardi‐Gascón et al. [20]). Classroom membership was also considered as a covariate because randomization was conducted at the classroom level.

### 2.4 Statistical Analysis

Separate multivariable linear regression models were estimated for each cognitive outcome. In cross‐sectional analyses, baseline cognitive outcomes were modeled as dependent variables, whereas baseline physical fitness indicators (cardiorespiratory fitness, handgrip strength, lower‐limb strength, and speed‐agility) were simultaneously entered as predictors together with age, sex, socioeconomic status, out‐of‐school physical activity, baseline height, baseline weight, and classroom membership as covariates. For longitudinal analyses, changes in physical fitness indicators were calculated as post‐intervention minus baseline values. For the 4 × 10‐m shuttle run, negative change values indicated improved speed‐agility performance. Longitudinal associations were examined using baseline‐adjusted analysis of covariance (ANCOVA) models implemented as multivariable linear regression. In these models, post‐intervention cognitive outcomes were modeled as dependent variables, while predictors included the corresponding baseline cognitive outcome, changes in physical fitness indicators, and the same covariates described above. Because only four classrooms were available, classroom membership was included as a fixed effect in the primary analyses, as random‐effects estimation with a very small number of clusters may produce unstable variance estimates. Intervention group was not entered simultaneously with classroom membership because group allocation was determined by classroom membership, which would have introduced collinearity. Sensitivity analyses were additionally performed using linear mixed‐effects models including classroom as a random intercept to examine whether the direction and pattern of the associations were consistent under an alternative clustering specification. Given the limited number of classroom clusters, these mixed‐effects analyses were considered exploratory sensitivity analyses supplementary to the primary baseline‐adjusted ANCOVA models. To reduce the risk of type I error due to multiple testing, false discovery rate (FDR) correction was applied using the Benjamini–Hochberg procedure separately across cross‐sectional and longitudinal analyses. Only FDR‐corrected *p* < 0.05 were considered statistically significant. Regression coefficients (*B*), 95% confidence intervals, and *p*‐values before and after FDR correction were calculated. Multicollinearity was evaluated using variance inflation factors, all of which were below 2. All statistical analyses were conducted using R software version 4.6.0 (R Foundation for Statistical Computing, Vienna, Austria).

## 3 Results

**FIGURE 1:**
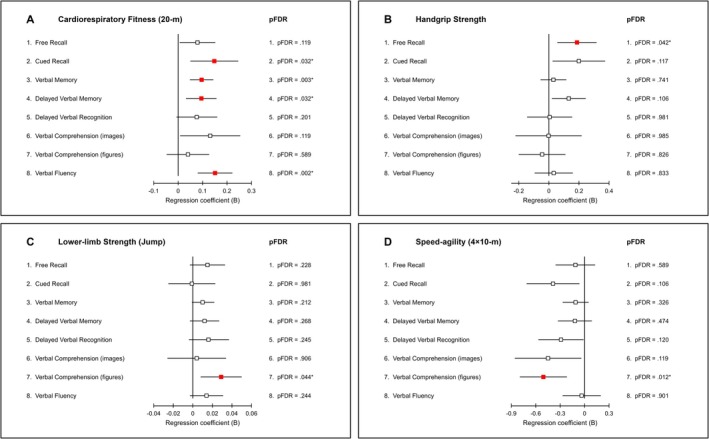
Cross‐sectional associations (expressed as regression coefficients) between physical fitness variables and cognitive outcomes. *Significant after FDR correction. Squares show regression coefficients (B) with 95% confidence intervals.

**FIGURE 2:**
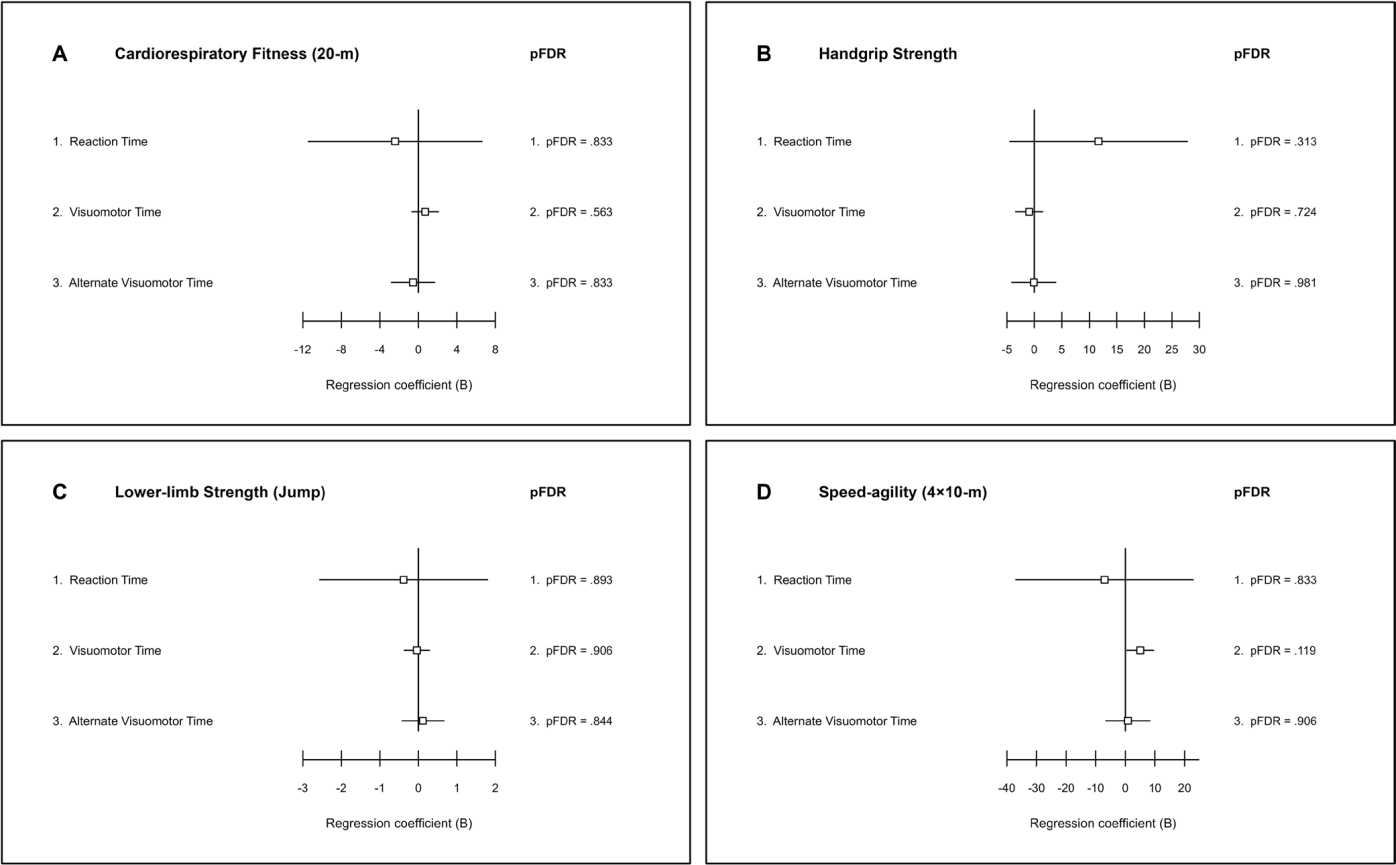
Cross‐sectional associations (expressed as regression coefficients) between physical fitness variables and cognitive time‐based outcomes. *Significant after FDR correction. Squares show regression coefficients (B) with 95% confidence intervals.

**FIGURE 3:**
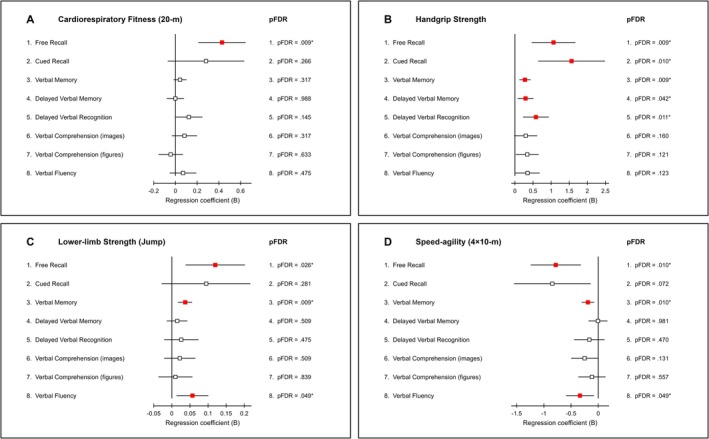
Longitudinal associations (expressed as regression coefficients) between physical fitness variables and cognitive outcomes. *Significant after FDR correction. Squares show regression coefficients (B) with 95% confidence intervals.

**FIGURE 4:**
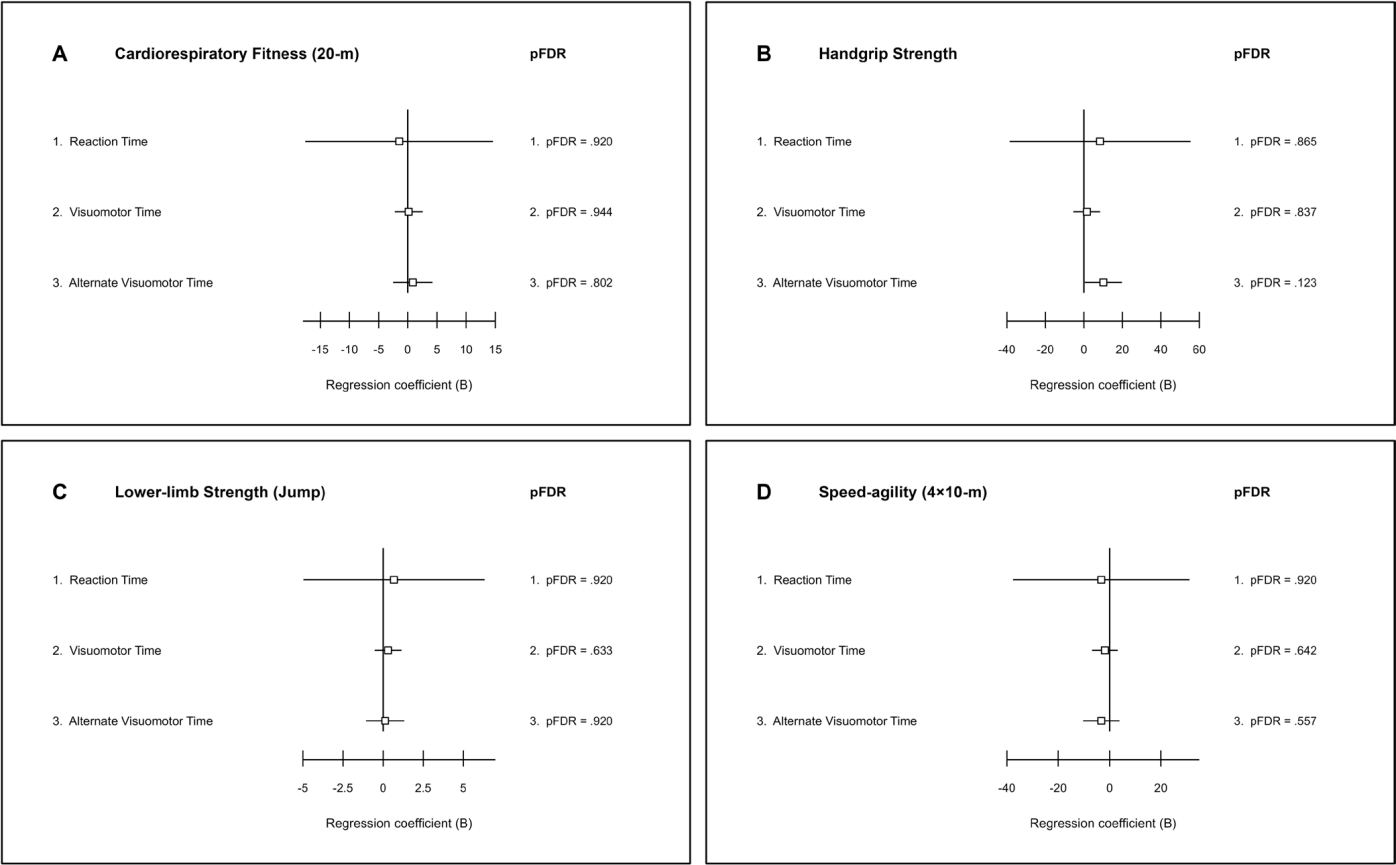
Longitudinal associations (expressed as regression coefficients) between physical fitness variables and cognitive time‐based outcomes. *Significant after FDR correction. Squares show regression coefficients (B) with 95% confidence intervals.

Out of a total of 99 children (58 girls and 41 boys), 48 were assigned to the control group, 26 to the LPA group, and 25 to the MVPA group (more details in Martin‐Martinez et al. [15]). The cross‐sectional and longitudinal associations between physical fitness indicators and cognitive performance are shown in Table 1 and Table 2, respectively, and are graphically summarized in Figures 1, 2, 3, 4. Specifically, Figures 1 and 2 display the cross‐sectional associations for cognitive performance and time‐based cognitive outcomes, respectively, whereas Figures 3 and 4 display the corresponding longitudinal associations.

**TABLE 1:** Cross‐sectional associations between physical fitness and cognitive performance.

Cognitive outcome	Cardiorespiratory fitness (20‐m)	Handgrip strength	Lower‐limb strength (jump)	Speed‐agility (4 × 10‐m)
Free Recall	*B* = 0.079 [0.006, 0.152] (*p* = 0.034; pFDR = 0.119)	***B* = 0.187 [0.056, 0.317]** **(*p* = 0.006; pFDR = 0.042)**	*B* = 0.015 [−0.003, 0.033] (*p* = 0.093; pFDR = 0.228)	*B* = −0.113 [−0.356, 0.129] (*p* = 0.356; pFDR = 0.589)
Cued Recall	***B* = 0.148 [0.050, 0.246]** **(*p* = 0.003; pFDR = 0.032)**	*B* = 0.199 [0.024, 0.374] (*p* = 0.027; pFDR = 0.117)	*B* = −0.001 [−0.025, 0.023] (*p* = 0.924; pFDR = 0.981)	*B* = −0.388 [−0.713, −0.063] (*p* = 0.020; pFDR = 0.106)
Verbal Memory	***B* = 0.096 [0.048, 0.144]** **(*p* = 0.0001; pFDR = 0.003)**	*B* = 0.030 [−0.056, 0.116] (*p* = 0.488; pFDR = 0.741)	*B* = 0.010 [−0.001, 0.022] (*p* = 0.082; pFDR = 0.212)	*B* = −0.111 [−0.270, 0.049] (*p* = 0.170; pFDR = 0.326)
Delayed Verbal Memory	***B* = 0.095 [0.032, 0.157]** **(*p* = 0.004; pFDR = 0.032)**	*B* = 0.132 [0.020, 0.245] (*p* = 0.022; pFDR = 0.106)	*B* = 0.012 [−0.003, 0.027] (*p* = 0.128; pFDR = 0.268)	*B* = −0.119 [−0.328, 0.089] (*p* = 0.259; pFDR = 0.474)
Delayed Verbal Recognition	*B* = 0.076 [−0.007, 0.160] (*p* = 0.073; pFDR = 0.201)	*B* = 0.005 [−0.145, 0.155] (*p* = 0.947; pFDR = 0.981)	*B* = 0.016 [−0.004, 0.037] (*p* = 0.111; pFDR = 0.245)	*B* = −0.290 [−0.568, −0.012] (*p* = 0.041; pFDR = 0.120)
Verbal Comprehension (images)	*B* = 0.131 [0.008, 0.254] (*p* = 0.038; pFDR = 0.119)	*B* = −0.002 [−0.222, 0.218] (*p* = 0.985; pFDR = 0.985)	*B* = 0.004 [−0.026, 0.034] (*p* = 0.790; pFDR = 0.906)	*B* = −0.449 [−0.857, −0.040] (*p* = 0.032; pFDR = 0.119)
Verbal Comprehension (figures)	*B* = 0.040 [−0.047, 0.127] (*p* = 0.361; pFDR = 0.589)	*B* = −0.045 [−0.201, 0.110] (*p* = 0.563; pFDR = 0.826)	***B* = 0.029 [0.008, 0.050]** **(*p* = 0.007; pFDR = 0.044)**	***B* = −0.506 [−0.795, −0.217]** **(*p* < 0.001; pFDR = 0.012)**
Verbal Fluency	***B* = 0.151 [0.080, 0.222]** **(*p* < 0.001; pFDR = 0.002)**	*B* = 0.032 [−0.095, 0.159] (*p* = 0.616; pFDR = 0.833)	*B* = 0.014 [−0.003, 0.031] (*p* = 0.105; pFDR = 0.244)	*B* = −0.037 [−0.271, 0.198] (*p* = 0.758; pFDR = 0.901)
Reaction Time	*B* = −2.410 [−11.477, 6.656] (*p* = 0.598; pFDR = 0.833)	*B* = 11.664 [−4.559, 27.887] (*p* = 0.156; pFDR = 0.313)	*B* = −0.381 [−2.577, 1.815] (*p* = 0.731; pFDR = 0.893)	*B* = −7.029 [−37.121, 23.062] (*p* = 0.643; pFDR = 0.833)
Visuomotor Time	*B* = 0.713 [−0.704, 2.130] (*p* = 0.320; pFDR = 0.563)	*B* = −0.944 [−3.480, 1.591] (*p* = 0.461; pFDR = 0.724)	*B* = −0.039 [−0.382, 0.305] (*p* = 0.823; pFDR = 0.906)	*B* = 5.062 [0.359, 9.766] (*p* = 0.035; pFDR = 0.119)
Alternate Visuomotor Time	*B* = −0.548 [−2.843, 1.746] (*p* = 0.636; pFDR = 0.833)	*B* = −0.106 [−4.211, 3.999] (*p* = 0.959; pFDR = 0.981)	*B* = 0.119 [−0.437, 0.675] (*p* = 0.671; pFDR = 0.844)	*B* = 0.854 [−6.761, 8.469] (*p* = 0.824; pFDR = 0.906)

*Note:* Data are shown as regression coefficients (*B*), 95% confidence intervals [CI], *p*‐values, and false discovery rate‐adjusted *p*‐values (pFDR). Cross‐sectional models were adjusted for age, sex, socioeconomic status, out‐of‐school physical activity levels, baseline anthropometric variables, and classroom membership. Lower values indicated better performance for the 4 × 10‐m shuttle run test (speed‐agility), reaction time, visuomotor time, and alternate visuomotor time, whereas higher values indicated better performance for all other outcomes. Statistically significant associations after FDR correction (pFDR < 0.05) are indicated in bold.

**TABLE 2:** Longitudinal associations between physical fitness and cognitive gains.

Cognitive outcome	Cardiorespiratory fitness (20‐m)	Handgrip strength	Lower‐limb strength (Jump)	Speed‐agility (4 × 10‐m)
Free Recall	***B* = 0.429 [0.211, 0.646]** **(*p* < 0.001; pFDR = 0.009)**	***B* = 1.067 [0.463, 1.671]** **(*p* < 0.001; pFDR = 0.009)**	***B* = 0.120 [0.038, 0.202]** **(*p* = 0.005; pFDR = 0.026)**	***B* = −0.781 [−1.243, −0.319]** **(*p* = 0.001; pFDR = 0.010)**
Cued Recall	*B* = 0.281 [−0.071, 0.633] (*p* = 0.121; pFDR = 0.266)	***B* = 1.565 [0.643, 2.487]** **(*p* = 0.001; pFDR = 0.010)**	*B* = 0.095 [−0.028, 0.217] (*p* = 0.134; pFDR = 0.281)	*B* = −0.844 [−1.551, −0.138] (*p* = 0.021; pFDR = 0.072)
Verbal Memory	*B* = 0.042 [−0.017, 0.102] (*p* = 0.166; pFDR = 0.317)	***B* = 0.277 [0.123, 0.431]** **(*p* = 0.001; pFDR = 0.009)**	***B* = 0.037 [0.017, 0.056]** **(*p* < 0.001; pFDR = 0.009)**	***B* = −0.189 [−0.303, −0.075]** **(*p* = 0.002; pFDR = 0.010)**
Delayed Verbal Memory	*B* = −0.001 [−0.080, 0.079] (*p* = 0.988; pFDR = 0.988)	***B* = 0.294 [0.077, 0.512]** **(*p* = 0.010; pFDR = 0.042)**	*B* = 0.015 [−0.014, 0.043] (*p* = 0.313; pFDR = 0.509)	*B* = −0.005 [−0.179, 0.170] (*p* = 0.959; pFDR = 0.981)
Delayed Verbal Recognition	*B* = 0.123 [−0.003, 0.249] (*p* = 0.059; pFDR = 0.145)	***B* = 0.580 [0.224, 0.935]** **(*p* = 0.002; pFDR = 0.011)**	*B* = 0.026 [−0.021, 0.074] (*p* = 0.281; pFDR = 0.475)	*B* = −0.163 [−0.444, 0.117] (*p* = 0.256; pFDR = 0.470)
Verbal Comprehension (images)	*B* = 0.083 [−0.033, 0.199] (*p* = 0.166; pFDR = 0.317)	*B* = 0.298 [−0.019, 0.616] (*p* = 0.069; pFDR = 0.160)	*B* = 0.022 [−0.021, 0.065] (*p* = 0.324; pFDR = 0.509)	*B* = −0.250 [−0.497, −0.003] (*p* = 0.050; pFDR = 0.131)
Verbal Comprehension (figures)	*B* = −0.044 [−0.157, 0.069] (*p* = 0.451; pFDR = 0.633)	*B* = 0.340 [0.023, 0.658] (*p* = 0.039; pFDR = 0.121)	*B* = 0.010 [−0.037, 0.057] (*p* = 0.687; pFDR = 0.839)	*B* = −0.116 [−0.367, 0.135] (*p* = 0.367; pFDR = 0.557)
Verbal Fluency	*B* = 0.070 [−0.054, 0.193] (*p* = 0.271; pFDR = 0.475)	*B* = 0.349 [0.013, 0.685] (*p* = 0.045; pFDR = 0.123)	***B* = 0.057 [0.013, 0.101]** **(*p* = 0.013; pFDR = 0.049)**	***B* = −0.336 [−0.593, −0.079]** **(*p* = 0.012; pFDR = 0.049)**
Reaction Time	*B* = −1.476 [−17.559, 14.606] (*p* = 0.858; pFDR = 0.920)	*B* = 8.376 [−38.621, 55.373] (*p* = 0.728; pFDR = 0.865)	*B* = 0.679 [−4.968, 6.326] (*p* = 0.814; pFDR = 0.920)	*B* = −3.212 [−37.545, 31.120] (*p* = 0.855; pFDR = 0.920)
Visuomotor Time	*B* = 0.151 [−2.231, 2.533] (*p* = 0.901; pFDR = 0.944)	*B* = 1.532 [−5.391, 8.456] (*p* = 0.666; pFDR = 0.837)	*B* = 0.315 [−0.518, 1.148] (*p* = 0.460; pFDR = 0.633)	*B* = −1.823 [−6.878, 3.233] (*p* = 0.482; pFDR = 0.642)
Alternate Visuomotor Time	*B* = 0.858 [−2.521, 4.237] (*p* = 0.620; pFDR = 0.802)	*B* = 10.124 [0.455, 19.793] (*p* = 0.043; pFDR = 0.123)	*B* = 0.133 [−1.050, 1.315] (*p* = 0.826; pFDR = 0.920)	*B* = −3.201 [−10.307, 3.905] (*p* = 0.380; pFDR = 0.557)

*Note:* Data are shown as regression coefficients (*B*), 95% confidence intervals [CI], *p*‐values, and false discovery rate‐adjusted *p*‐values (pFDR). Longitudinal analyses were adjusted for baseline cognitive performance, age, sex, socioeconomic status, physical activity levels, baseline anthropometric variables, and classroom membership. Lower values indicated better performance for the 4 × 10‐m shuttle run test (speed‐agility), reaction time, visuomotor time, and alternate visuomotor time, whereas higher values indicated better performance for all other outcomes. Statistically significant associations after FDR correction (pFDR < 0.05) are indicated in bold.

### 3.1 Cross‐Sectional Associations

#### 3.1.1 Cardiorespiratory Fitness

In cross‐sectional analyses at baseline, cardiorespiratory fitness showed significant positive associations with verbal memory (pFDR = 0.003), delayed verbal memory (pFDR = 0.032), and cued recall (pFDR = 0.032), although no associations were observed with free recall. Regarding language outcomes, cardiorespiratory fitness was associated with verbal fluency (pFDR = 0.002). No significant associations were observed with visuomotor or executive outcomes, including reaction time, visuomotor, and alternate visuomotor tasks.

#### 3.1.2 Handgrip Strength

Handgrip strength was positively associated with free recall performance (pFDR = 0.042). No other significant cross‐sectional associations were observed for memory, language, verbal comprehension, reaction time, or visuomotor and alternate visuomotor tasks.

#### 3.1.3 Speed‐Agility

Better speed‐agility performance, indicated by lower 4 × 10‐m shuttle‐run time, was independently associated with verbal comprehension outcomes. Specifically, better speed‐agility was significantly associated with verbal comprehension (figures) (pFDR = 0.012) after FDR correction, whereas no associations were observed for verbal comprehension (images). No other significant cross‐sectional associations were observed for memory, language, reaction time, or visuomotor and alternate visuomotor tasks.

#### 3.1.4 Lower‐Limb Strength

Lower‐limb strength was positively associated with verbal comprehension (figures) (pFDR = 0.044) after FDR correction, whereas no associations were observed for verbal comprehension (images). For other domains, lower‐limb strength was not associated with memory, language, reaction time, or visuomotor and alternate visuomotor tasks.

### 3.2 Longitudinal Associations

#### 3.2.1 Cardiorespiratory Fitness

Over time, changes in cardiorespiratory fitness were significantly associated with longitudinal gains in free recall performance (pFDR = 0.009). However, they were not associated with changes in cued recall, verbal memory, or delayed verbal memory outcomes. No significant longitudinal associations were observed for verbal comprehension, verbal fluency, reaction time, or visuomotor and alternate visuomotor tasks.

#### 3.2.2 Handgrip Strength

Changes in handgrip strength were longitudinally associated with changes in free recall (pFDR = 0.009), cued recall (pFDR = 0.010), verbal memory (pFDR = 0.009), delayed verbal memory (pFDR = 0.042), and delayed verbal memory recognition (pFDR = 0.011). Beyond memory, no significant longitudinal associations were observed for verbal comprehension, verbal fluency, reaction time, or visuomotor and alternate visuomotor tasks.

#### 3.2.3 Speed‐Agility

Changes in speed‐agility were associated with selected memory‐related outcomes. Specifically, improvements in speed‐agility performance were longitudinally associated with improvements in free recall (pFDR = 0.010) and verbal memory (pFDR = 0.010). However, no significant longitudinal associations were observed for cued recall or delayed verbal memory. Regarding language, significant longitudinal associations were observed for verbal fluency (pFDR = 0.049). No significant associations were observed for verbal comprehension (images nor figures), reaction time, or visuomotor and alternate visuomotor tasks. For the 4 × 10‐m speed‐agility test, lower values indicate better performance.

#### 3.2.4 Lower‐Limb Strength

Changes in lower‐limb strength were associated with selected memory‐related outcomes. Specifically, improvements in lower‐limb strength performance were longitudinally associated with improvements in free recall (pFDR = 0.026) and verbal memory (pFDR = 0.009). However, no significant longitudinal associations were observed for cued recall or delayed verbal memory. In language, significant longitudinal associations were observed for verbal fluency (pFDR = 0.049). No significant associations were observed for verbal comprehension (images nor figures), reaction time, or visuomotor and alternate visuomotor tasks.

### 3.3 Sensitivity Analyses

Sensitivity analyses using linear mixed‐effects models including classroom as a random intercept showed broadly similar directions of association for several memory‐related outcomes, particularly for handgrip strength. However, estimates were less precise, and none of these associations survived FDR correction in the mixed‐effects sensitivity models. Therefore, these analyses should be interpreted as providing directional consistency with the primary ANCOVA models rather than confirmatory evidence of robustness. Exploratory visuomotor error outcomes were additionally examined in the supplementary mixed‐effects analyses. Detailed mixed‐effects model results are presented in Table S1.

## 4 Discussion

The present study examined cross‐sectional and longitudinal associations between physical fitness components and cognitive performance in preschool children. Overall, the findings suggest that fitness–cognition relationships during early childhood may be selective rather than generalized across cognitive domains. The most consistent associations were observed for memory‐ and language‐related outcomes, whereas reaction time and visuomotor measures showed non‐significant associations after FDR correction. Longitudinal analyses particularly highlighted handgrip strength associations with memory‐related outcomes, whereas cardiorespiratory fitness, speed‐agility, and lower‐limb strength showed more selective associations across specific cognitive domains.

These results align with previous cross‐sectional studies in preschool populations. Zhou et al. [9] reported that higher overall fitness was associated with better executive functioning, and Li et al. [12] found that agility and coordination related to attentional control in preschool children. Similarly, Geertsen et al. [21] observed associations between agility and executive tasks in school‐aged children (8–10 years). Our findings partially align with these previous studies, although the observed associations were more selective than generalized across all cognitive domains. Divergences may reflect developmental timing, since certain executive skills mature later, or potential measurement limitations in tasks adapted for very young children. This selective pattern supports recent conceptual perspectives proposing that movement‐related cognitive effects may depend not only on exercise exposure itself, but also on whether the movement context engages domain‐general or domain‐specific executive processes [22]. From this perspective, cognitively engaging movement contexts may preferentially influence selected cognitive functions depending on task demands and contextual alignment between movement and cognition. It is worth noting that the present results were derived from a trial in which a cognitively engaging intervention was performed, with students working on language‐related outcomes while they performed physical activity. This can have a relevant impact on the observed findings, and it is possible that different cognitive effects would have been observed with other types of physical activity. Importantly, although sedentary behavior was not directly examined in the present study, interventions aimed at improving fitness and cognitive function in early childhood may benefit not only from promoting physical activity, but also from reducing or replacing sedentary behaviors that are less cognitively engaging. In this context, it is also relevant to consider that sedentary behavior may influence different aspects of brain health. Importantly, sedentary behavior should not be considered a unitary construct, as its associations with brain health may depend on contextual and cognitive characteristics, including the degree of mental engagement and content relevance [23, 24].

Our longitudinal analyses extend prior evidence by showing that changes in fitness, rather than baseline levels alone, are significantly associated with cognitive improvements. Reisberg et al. [25] demonstrated that muscular strength in early childhood forecasts later cognitive skills, a finding echoed here through the role of handgrip strength. Likewise, improvements in cardiorespiratory fitness were selectively associated with free recall performance, whereas handgrip strength showed the most consistent longitudinal associations with memory‐related outcomes. These findings align with previous literature suggesting that fitness–cognition relationships may vary according to the specific cognitive domain assessed and developmental stage [26, 27]. By contrast, we observed null associations with visuomotor control and reaction time, which may be due to immature developmental trajectories of these functions at ages 4–5.

The biological plausibility of these associations is supported by broader neurodevelopmental frameworks suggesting that physical activity and fitness may influence brain health through pathways involving neuroplasticity, cerebral blood flow, and neurotrophic factors. However, direct neurobiological evidence in preschool populations remains limited. Previous literature suggests that aerobic fitness may be associated with mechanisms linked to hippocampal development through increased neurogenesis and BDNF expression, thereby enhancing memory [27]. Muscular strength could contribute through proprioceptive feedback and motor planning, processes linked to information encoding and retrieval [5, 6]. Although the mechanisms underlying fitness–cognition associations during early childhood remain incompletely understood, previous studies suggest that physical activity and fitness may influence neurodevelopment through pathways involving neuroplasticity, cerebral blood flow, and neurotrophic factors such as BDNF. However, direct neurobiological evidence in preschool populations remains limited, and future studies incorporating neuroimaging and physiological measures are needed. Previous neuroimaging studies in children have reported associations between higher fitness levels and structural or functional markers of brain health, including regions involved in memory and executive function [5, 27].

Strengths of this study include the integration of cross‐sectional and longitudinal analyses, the use of computerized cognitive performance measures and objective physical fitness assessments, and the focus on a critical developmental window such as preschool age. Nevertheless, some limitations warrant consideration. The sample size was modest, which may have constrained statistical power. Residual confounding cannot be excluded, as factors such as home environment or parental education were not fully captured. In addition, direct validation evidence for the complete BENCI battery in Spanish‐speaking preschool children aged 3–6 years remains limited. Although the selected subtests were feasible and developmentally appropriate in the present sample, future studies should further establish their reliability, validity, and sensitivity specifically in preschool populations. Importantly, the BENCI‐derived outcomes should be interpreted as standardized computerized cognitive performance measures rather than as fully validated neuropsychological diagnostic measures for preschool children. Furthermore, the small number of classrooms (*n* = 4) limits the robustness of cluster‐level inference, even after adjustment for classroom membership and sensitivity analyses. Therefore, longitudinal findings should be interpreted cautiously. Additionally, although mixed‐effects sensitivity analyses showed broadly similar directions for several associations, no association survived FDR correction in these models; therefore, these analyses do not fully resolve the limitations related to cluster‐level inference. Another limitation is that the present secondary analyses were conducted within a trial in which physical activity was integrated into foreign‐language lessons. Therefore, the observed associations with memory‐ and language‐related outcomes may partly reflect the cognitively engaging and language‐specific context of the intervention, as well as shared responsiveness to the intervention, rather than isolated associations with changes in physical fitness. Future studies should employ larger samples, refine cognitive assessments for this age group, and incorporate neuroimaging or molecular biomarkers to clarify mechanisms. Randomized controlled trials targeting specific fitness components are also needed to establish causality.

From a practical perspective, our findings support the potential relevance of structured physical activity in early education. Integrating activities that promote muscular strength and aerobic capacity may yield benefits that extend beyond physical health, fostering memory, language, and foundational executive skills. Importantly, the cognitive effects of movement may depend not only on activity quantity or intensity, but also on the cognitive, coordinative, and social demands embedded within the movement context. Previous literature suggests that cognitively engaging or open‐skill movement contexts may confer stronger cognitive benefits than more repetitive or closed‐skill activities, particularly for executive‐function‐related outcomes [26]. Approaches that combine movement with cognitive engagement should therefore be considered a promising hypothesis for future preschool interventions, aligning with whole‐child models of preschool education.

## 5 Perspective

Future research should move beyond global movement–cognition hypotheses and examine which types of movement, delivered in which contexts, are most strongly associated with specific cognitive domains during early childhood. Emerging conceptual frameworks suggest that these associations may depend not only on physical activity quantity or intensity, but also on the cognitive, coordinative, and social demands embedded within movement contexts [22, 26]. Therefore, future studies should explore whether cognitively engaging movement interventions, open‐skill activities, or integrated movement–learning approaches differentially influence domain‐general and domain‐specific executive processes in preschool children.

Additional longitudinal and experimental studies with larger and more diverse preschool samples are needed to clarify the mechanisms underlying fitness–cognition associations during early childhood. Future research would also benefit from incorporating validated preschool cognitive assessments, objective assessments of physical activity and sedentary behavior, neurophysiological or neuroimaging measures, and multi‐level analytical approaches accounting for school and classroom membership. Moreover, studies should examine whether movement–cognition relationships differ according to developmental stage, socioeconomic background, neurodevelopmental profile, or educational context. Better understanding these factors may help optimize future school‐based interventions aimed at supporting both physical and cognitive development during this sensitive developmental period [8, 23].

## Author Contributions

**Carlos Martin‐Martinez:** conceptualization, methodology, formal analysis, investigation, writing – original draft preparation. **Pedro L. Valenzuela:** conceptualization, methodology, formal analysis, data curation, writing – review and editing. **Asier Mañas:** conceptualization, methodology, formal analysis, investigation, data curation, writing – review and editing, supervision. **Oscar Martinez‐de‐Quel:** conceptualization, methodology, formal analysis, investigation, data curation, writing – review and editing, supervision. All authors revised the manuscript for important intellectual content, approved the final manuscript as submitted and agreed to be accountable for all aspects of the work.

## Funding

Research by PLV is supported by a postdoctoral contract granted by Agencia Estatal de Investigación (RYC2024‐048275‐I). This work was conducted during A.M.'s research stay at the Department of Didactics of Language, Arts and Physical Education (University Complutense of Madrid) and the Center UCM‐ISCIII for Human Evolution and Behavior (Madrid, Spain). This stay was supported by a “Margarita Salas” Requalification contract (MS2021) funded by the University of Castilla‐La Mancha. Funding for open access charge: Universidad de Huelva/CBUA. No specific funding was received for this study.

## Ethics Statement

Ethical approval was obtained from the Research Ethics Committee at Universidad Complutense de Madrid (CE_20211118‐16_SAL).

## Conflicts of Interest

The authors declare no conflicts of interest. There was no external funding (apart from the author's salary in their institutions), and therefore funders had no role in (1) study design; (2) the collection, analysis, and interpretation of data; (3) the writing of the report; nor on (4) the decision to submit the manuscript for publication.

## Supporting information


**Table S1:** Exploratory sensitivity analyses using linear mixed‐effects models including classroom as a random intercept.

## Data Availability

The data that support the findings of this study are available from the corresponding author upon reasonable request.
