# Supplementary material for: Physical Fitness and Cognitive Performance in Preschool Children: Cross‐Sectional and Longitudinal Associations From the ELFIT Trial
Source: Scand J Med Sci Sports. 2026 Jun 22;36(6):e70325. doi: 10.1111/sms.70325 (PMC13287308; doi:10.1111/sms.70325)
Supplement: Supplementary file 1 — Table S1: Exploratory sensitivity analyses using linear mixed‐effects models including classroom as a random intercept. [file SMS-36-e70325-s001.docx]

**Supplementary Table S1. Exploratory sensitivity analyses using linear mixed-effects models including classroom as a random intercept**

| **Cognitive Outcome** | **Cardiorespiratory Fitness (20-m)** | **Handgrip Strength** | **Lower-limb Strength (Jump)** | **Speed-agility (4×10-m)** |
| --- | --- | --- | --- | --- |
| **Free Recall** | B = 0.163 [−0.010, 0.336]  (p = .069; pFDR = .299) | B = 0.607 [0.168, 1.046]  (p = .008; pFDR = .070) | B = 0.047 [−0.015, 0.108]  (p = .140; pFDR = .455) | B = −0.297 [−0.652, 0.058]  (p = .105; pFDR = .390) |
| **Cued Recall** | B = −0.180 [−0.460, 0.100]  (p = .212; pFDR = .525) | B = 0.998 [0.286, 1.710]  (p = .007; pFDR = .070) | B = −0.001 [−0.096, 0.095]  (p = .989; pFDR = .989) | B = −0.104 [−0.670, 0.461]  (p = .718; pFDR = .883) |
| **Verbal Memory** | B = 0.017 [−0.041, 0.075]  (p = .566; pFDR = .815) | B = 0.206 [0.057, 0.356]  (p = .008; pFDR = .070) | B = 0.027 [0.008, 0.047]  (p = .008; pFDR = .070) | B = −0.158 [−0.268, −0.048]  (p = .006; pFDR = .070) |
| **Delayed Verbal Memory** | B = 0.008 [−0.072, 0.088]  (p = .843; pFDR = .932) | B = 0.314 [0.103, 0.526]  (p = .004; pFDR = .070) | B = 0.017 [−0.011, 0.045]  (p = .235; pFDR = .545) | B = −0.024 [−0.199, 0.151]  (p = .791; pFDR = .914) |
| **Delayed Verbal Recognition** | B = 0.084 [−0.046, 0.214]  (p = .210; pFDR = .525) | B = 0.475 [0.125, 0.826]  (p = .009; pFDR = .070) | B = 0.013 [−0.034, 0.060]  (p = .595; pFDR = .815) | B = −0.087 [−0.372, 0.198]  (p = .552; pFDR = .815) |
| **Verbal Comprehension (Images)** | B = 0.062 [−0.055, 0.179]  (p = .303; pFDR = .583) | B = 0.187 [−0.130, 0.503]  (p = .251; pFDR = .545) | B = 0.001 [−0.043, 0.046]  (p = .957; pFDR = .975) | B = −0.215 [−0.462, 0.031]  (p = .090; pFDR = .361) |
| **Verbal Comprehension (Figures)** | B = −0.066 [−0.182, 0.051]  (p = .273; pFDR = .545) | B = 0.302 [−0.010, 0.614]  (p = .061; pFDR = .289) | B = −0.009 [−0.057, 0.039]  (p = .716; pFDR = .883) | B = −0.110 [−0.367, 0.147]  (p = .403; pFDR = .676) |
| **Verbal Fluency** | B = 0.025 [−0.102, 0.152]  (p = .701; pFDR = .883) | B = 0.253 [−0.077, 0.584]  (p = .136; pFDR = .455) | B = 0.045 [0.000, 0.090]  (p = .052; pFDR = .270) | B = −0.276 [−0.539, −0.013]  (p = .043; pFDR = .246) |
| **Reaction Time** | B = −1.181 [−17.092, 14.731]  (p = .885; pFDR = .958) | B = 9.702 [−35.692, 55.096]  (p = .676; pFDR = .883) | B = 0.809 [−4.737, 6.355]  (p = .776; pFDR = .914) | B = −3.743 [−37.727, 30.241]  (p = .830; pFDR = .932) |
| **Visuomotor Errors** | B = −0.244 [−0.674, 0.185]  (p = .268; pFDR = .545) | B = −0.486 [−1.717, 0.746]  (p = .442; pFDR = .718) | B = −0.107 [−0.254, 0.041]  (p = .161; pFDR = .492) | B = 0.613 [−0.301, 1.528]  (p = .192; pFDR = .525) |
| **Visuomotor Time** | B = 0.117 [−2.288, 2.522]  (p = .925; pFDR = .962) | B = 1.888 [−4.827, 8.604]  (p = .583; pFDR = .815) | B = 0.384 [−0.438, 1.206]  (p = .363; pFDR = .650) | B = −1.790 [−6.863, 3.282]  (p = .491; pFDR = .774) |
| **Alternate Visuomotor Errors** | B = −0.304 [−0.837, 0.229]  (p = .267; pFDR = .545) | B = 0.661 [−0.874, 2.196]  (p = .401; pFDR = .676) | B = 0.123 [−0.063, 0.308]  (p = .199; pFDR = .525) | B = 0.069 [−1.067, 1.205]  (p = .906; pFDR = .961) |
| **Alternate Visuomotor Time** | B = 1.008 [−2.334, 4.350]  (p = .556; pFDR = .815) | B = 10.450 [1.088, 19.811]  (p = .031; pFDR = .204) | B = 0.205 [−0.957, 1.368]  (p = .730; pFDR = .883) | B = −3.524 [−10.535, 3.487]  (p = .327; pFDR = .608) |

Sensitivity analyses were conducted using linear mixed-effects models including classroom as a random intercept. Data are shown as regression coefficients (B), 95% confidence intervals [CI], p-values, and false discovery rate-adjusted p-values (pFDR). Models were adjusted for age, sex, socioeconomic status, physical activity levels, baseline anthropometric variables, and baseline cognitive performance. Lower values indicated better performance for the 4×10-m shuttle run test (speed-agility), reaction time, visuomotor tasks, and alternate visuomotor tasks, whereas higher values indicated better performance for all other outcomes. Supplementary analyses additionally included exploratory visuomotor error outcomes. Associations with pFDR < .05 were considered statistically significant. These sensitivity analyses should be interpreted as exploratory and not as confirmatory evidence of robustness.
